# Volatiles from soil‐borne fungi affect directional growth of roots

**DOI:** 10.1111/pce.13890

**Published:** 2020-09-30

**Authors:** Kay Moisan, Jos M. Raaijmakers, Marcel Dicke, Dani Lucas‐Barbosa, Viviane Cordovez

**Affiliations:** ^1^ Laboratory of Entomology Wageningen University and Research Wageningen The Netherlands; ^2^ Department of Microbial Ecology Netherlands Institute of Ecology Wageningen The Netherlands; ^3^ Institute of Biology Leiden University Leiden The Netherlands; ^4^ Present address: Bio‐communication & Ecology ETH Zürich Zürich Switzerland

**Keywords:** chemotaxis, fungal volatiles, *Rhizoctonia solani*, root attraction, root olfactometer, root pathogen

## Abstract

Volatiles play major roles in mediating ecological interactions between soil (micro)organisms and plants. It is well‐established that microbial volatiles can increase root biomass and lateral root formation. To date, however, it is unknown whether microbial volatiles can affect directional root growth. Here, we present a novel method to study belowground volatile‐mediated interactions. As proof‐of‐concept, we designed a root Y‐tube olfactometer, and tested the effects of volatiles from four different soil‐borne fungi on directional growth of *Brassica rapa* roots in soil. Subsequently, we compared the fungal volatile organic compounds (VOCs) previously profiled with Gas Chromatography–Mass Spectrometry (GC–MS). Using our newly designed setup, we show that directional root growth in soil is differentially affected by fungal volatiles. Roots grew more frequently toward volatiles from the root pathogen *Rhizoctonia solani*, whereas volatiles from the other three saprophytic fungi did not impact directional root growth. GC–MS profiling showed that six VOCs were exclusively emitted by *R. solani.* These findings verify that this novel method is suitable to unravel the intriguing chemical cross‐talk between roots and soil‐borne fungi and its impact on root growth.

## 1 INTRODUCTION

Roots represent a vital plant organ that ensures anchoring in the soil and mechanical support, as well as water and nutrient acquisition (Erktan, McCormack, & Roumet, 2018). The rhizosphere, that is the narrow zone of soil surrounding and influenced by roots, harbours dynamic communities of microorganisms that exploit root exudates as food source (Hassan, McInroy, & Kloepper, 2019; Philippot, Raaijmakers, Lemanceau, & van der Putten, 2013). Root exudates, including volatiles, can recruit beneficial microorganisms from a distance to enhance plant performance (el Zahar Haichar, Santaella, Heulin, & Achouak, 2014; Rasmann et al., 2005; Schulz‐Bohm et al., 2018). However, plant pathogens, plant‐parasitic plants and nematodes can also exploit root volatiles to locate their host plants (Rasmann, Hiltpold, & Ali, 2012; Ruyter‐Spira et al., 2013).

Soil microorganisms also produce a large array of volatiles that can promote plant growth and affect root architecture, for example, shortening of the primary roots and enhancing lateral root formation (Casarrubia et al., 2016; Garnica‐Vergara et al., 2015; Moisan et al., 2019; Piechulla, Lemfack, & Kai, 2017). To date, however, it remains unknown whether microbial volatiles can affect directional root growth. Considering that plant pathogenic and non‐pathogenic soil‐borne microorganisms emit distinct volatile blends (Moisan et al., 2019; Müller et al., 2013), plants may be able to differentially respond to volatiles from pathogenic and non‐pathogenic beneficial microorganisms. In particular, roots are expected to grow away from soil‐borne pathogens. To investigate whether volatiles from soil‐borne fungi affect root biomass and root directional growth in soil, we designed a root Y‐tube olfactometer, providing roots of *Brassica rapa* with the choice to grow toward one volatile source: either toward volatiles emitted by the soil‐borne fungus or toward volatiles from the agar medium used for fungal growth (i.e. the control).

## 2 MATERIALS AND METHODS

### 2.1 Plant material and fungi

*Brassica rapa* L. (Brassicaceae), known as wild turnip, is an annual plant with a non‐tuberous tap root. The accession used in this study originated from a wild *B. rapa* population in Maarssen, The Netherlands (Danner et al., 2015). Seeds were surface‐sterilised with chlorine gas as previously described by Cordovez et al. (2017). The four soil‐borne fungi used in this study were *Fusarium oxysporum* f. sp. *raphani* (WCS600), *Rhizoctonia solani* AG2‐2 IIIb, *Chaetomium indicum* (CBS 356.92) and *Trichoderma viride* (CBS 101227). We previously showed that volatiles emitted by these fungi differentially affected *Arabidopsis thaliana* growth in vitro (Moisan et al., 2019). Both *C. indicum* and *T. viride* can colonise the rhizosphere or endosphere of roots of Brassicaceae (Junker, Draeger, & Schulz, 2012), whereas *F. oxysporum* and *R. solani* can be pathogenic to some brassicaceous species (Leeman et al., 1995; Pannecoucque & Höfte, 2009). To test their pathogenicity on *B. rapa*, we inoculated 1‐week‐old seedlings with these fungi (Data S1). The results of these bioassays showed that *R. solani* was indeed pathogenic to *B. rapa* with the typical root lesions and spear‐tip symptoms and a concomitant reduction in plant biomass. For the other three fungi, no disease symptoms nor adverse effects on root or shoot biomass of *B. rapa* were observed (Figure S1). All fungi were cultured on 1/5^th^ strength Potato Dextrose Agar (1/5^th^ PDA) at 25°C.

### 2.2 Root Y‐tube olfactometer assay

**FIGURE 1:**
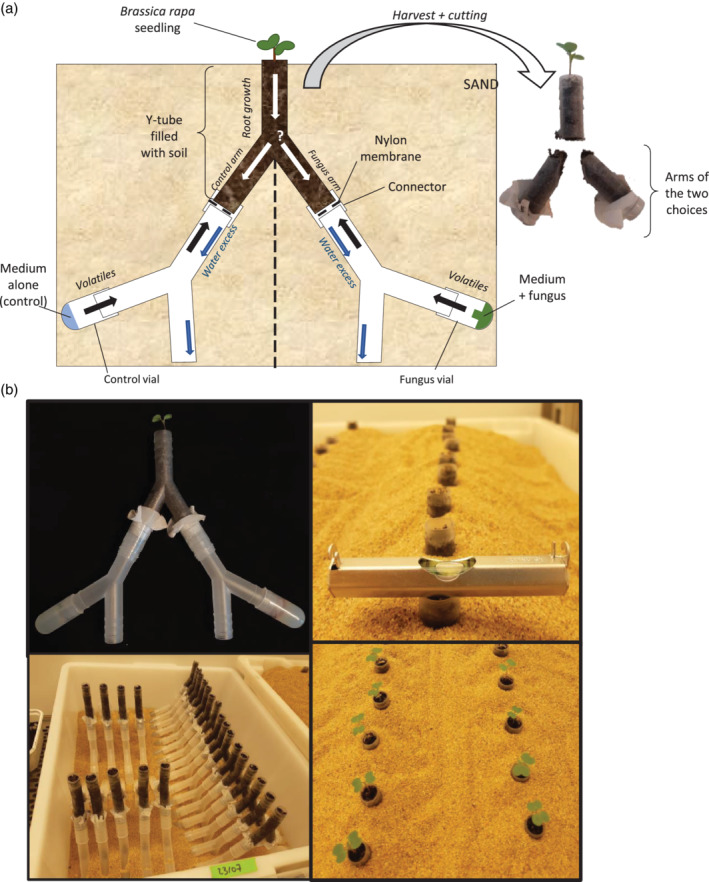
Illustration of the root Y‐tube olfactometer set‐up (a) Schematic representation of the olfactometer. One *Brassica rapa* seed was sown on the top of the Y‐tube containing soil, and primary root of the germinated seedling was “offered the choice” to grow into one of the arms of the Y‐tube: either toward the volatiles emitted by the test fungus on agar or toward the volatiles from the agar medium only, that is, the control. (b) Photographs of the root Y‐tube olfactometers during the experiment. Each olfactometer was buried in a box filled with sand, and kept in a growth cabinet for 7 days. At the harvest, the Y‐tubes were cut into three parts. Photo credits: Kay Moisan

To expose the roots to the fungal volatiles, we designed a Y‐tube olfactometer. The Y‐tube olfactometer consisted of a Y‐shaped plastic tube (Bürkle GmbH, Bad Bellingen, Germany) filled with a mixture of gamma‐irradiated potting soil and sand (1:1 v:v potting soil and sand, ø 2 mm sieved), with the two arms pointing downwards. Each arm was connected to an empty tube which was connected to a vial (bottom of 13 ml screw cap tube, Sarstedt AG & Co. KG, Nümbrecht, Germany) containing agar medium with or without one of the four fungi (Figure 1a). Prior to inoculation, these vials were surface‐sterilized with 70% ethanol in a flow cabinet, rinsed with ample sterile water, and dried by evaporation and kept under UV light for 45 min. In each vial, 3 ml of 1/5^th^ PDA medium was poured, and the fungi were inoculated on the same day as the sowing of the seeds. Tubes connecting the vials to the arms of the Y‐tube had an opening in the middle to allow excess water to flow out. Also, a nylon membrane (1 μm mesh size, Plastok associates Ltd., Birkenhead Wirral, UK) was placed between these tubes and the arms of the Y‐tube to allow exchange of volatiles.

After assembling all tubes together, sterile seeds were sown on the soil surface on top of the Y‐tubes. Primary roots of the germinated seedlings could grow downwards into one of the two arms of the Y‐tube: either toward the volatiles emitted by the test fungus or toward the volatiles from the agar medium only, that is, the control. The four fungi were individually tested against the control, and as a negative control we also tested control against control. One seed was sown per olfactometer and each fungal species was replicated in 30 independent root olfactometers. To mimic natural darkness conditions for the fungi and roots growing in the soil, the assembled root olfactometers were buried in a box filled with sand and placed in a growth cabinet (21°C; 16:8 h L:D; 70% RH). Positioning of the olfactometers followed a random complete block design, and the position of the fungus and control sides was alternated every two replicates. To avoid geotropism/gravity bias, each olfactometer was checked for horizontality with a level bubble measuring tool (Figure 1b). Plants were harvested 7 days after sowing to focus on the “choice” of the primary roots and to prevent growth of lateral roots in the two arms of the Y‐tube. Y‐tubes were cut with a hot blade to separate the two arms of the Y‐tube (Figure 1a). Roots were collected from both arms of the Y‐tube and washed to remove soil particles. Length of the primary roots was measured, and all roots were dried at 70°C for 5 days and weighed. Replicates with non‐germinated seeds or replicates where the control agar medium developed microbial contamination were discarded. The “choice” of the primary roots (i.e. arm of the Y‐tube in which the primary root grew) were analysed with binomial tests, and frequencies of root growth toward fungal volatiles were compared between the four fungi using a χ^2^ test (*H*_*0*_ = 0.50). Differences in root dry weights between control and fungal volatile‐exposed plants were analysed with paired‐sample Student *t*‐tests (*α* = 0.05), and differences in leaf dry weights upon choice of the primary root were analysed with Student *t*‐tests (*α* = 0.05).

### 2.3 Analysis of fungal volatile organic compounds

To compare the volatile organic compound (VOC) profiles of the four fungi, we statistically analysed VOC blends that were previously profiled as part of a larger number of soil‐borne fungi (Moisan et al., 2019). In brief, we employed a dynamic headspace sampling for 2 hours to collect VOCs from each of the fungi growing individually in glass Petri dishes containing 1/5^th^ PDA medium. VOC samples were analysed by GC–MS. Total Ion Chromatograms were used to generate values for peak areas, and the VOC profiles of the four selected fungi were analysed through a Projection to Latent Structures Discriminant Analysis (PLS‐DA) (SIMCA 15, Umetrics AB, Umeå, Sweden). This model was evaluated using a sevenfold cross‐validation test (*N* = 200; One‐way ANOVA) and with R^2^ and Q^2^ estimates.

## 3 RESULTS AND DISCUSSION

**FIGURE 2:**
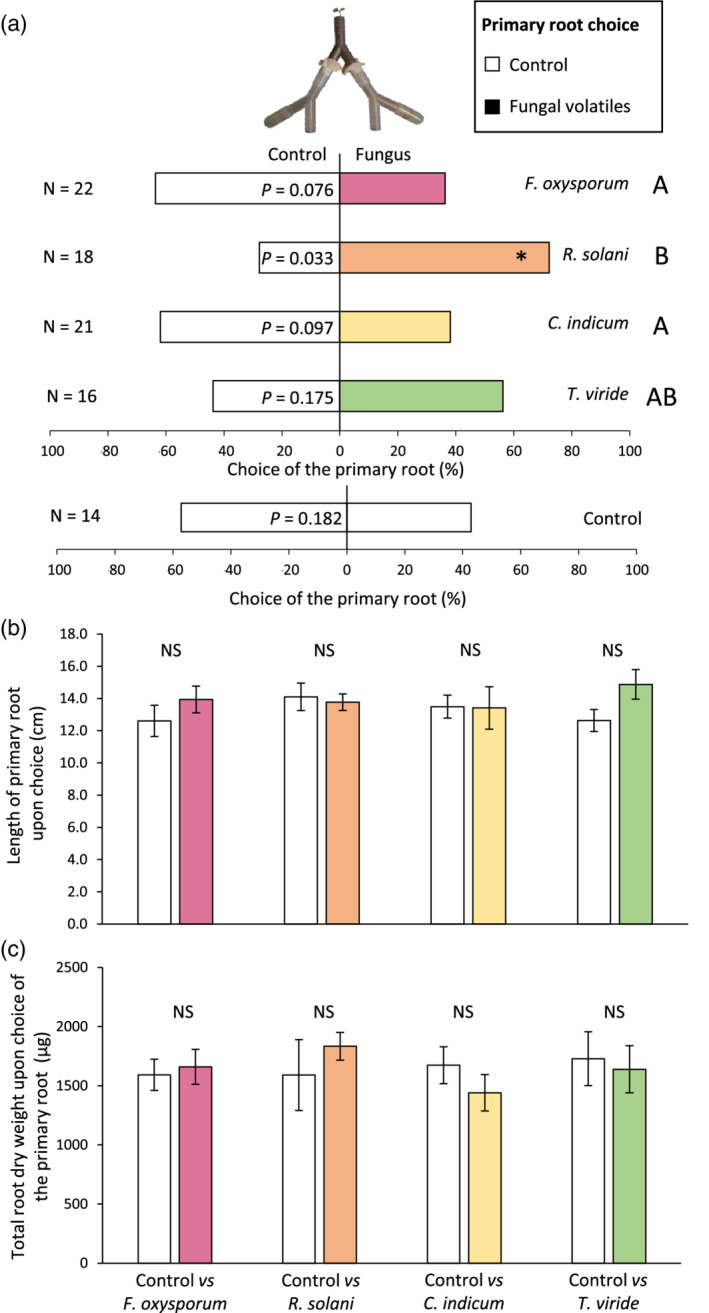
Effects of fungal volatiles on root growth (a) Percentages of *Brassica rapa* primary roots growing into the control arm (i.e. toward volatiles of the agar medium) or into the fungus arm (i.e. toward fungal volatiles) 7 days after sowing. Control versus control was used as a negative control. “*N*” indicates the number of root olfactometer replicates. Choices of primary roots were analysed with binomial tests (*H*_*0*_ = 0.50), and frequencies of choices toward the fungal volatiles between the four different two‐choices combinations were compared using χ^2^ tests. Uppercase letters indicate pairwise differences between control‐versus*‐*fungus choices. (b) Length (mean ± *SE*) of the primary root and (c) total root dry weight (mean ± *SE*) upon choice of the primary root toward the control or the fungal volatiles. Differences in primary root length and root dry weight were analysed with Student *t*‐tests (NS: *p* > .050) [Colour figure can be viewed at wileyonlinelibrary.com]

Fungal volatiles differentially affected directional growth of *B. rapa* roots (Figure 2a). Primary roots grew more frequently in the arm toward volatiles from *R. solani* than in the arm toward volatiles from the agar medium control (Figure 2a; *Obs.Freq* = 0.72; *p* = .033). In contrast, root directional growth was not significantly affected by the other three fungi (Figure 2a; *F. oxysporum*: *Obs.Freq* = 0.64; *p* = .076; *C. indicum: Obs.Freq* = 0.62; *p* = .097; *T. viride; Obs.Freq* = 0.56; *p* = .175). These results suggest that *B. rapa* roots sense and respond to volatile blends emitted by specific soil‐borne fungi. Interestingly, directional growth was significantly affected but length of the primary roots (Figure 2b; Student *t‐*tests; *p* > .05) and total root dry weight (Figure 2c; Student *t‐*tests; *p* > .05) were not impacted by the “choice” of the primary roots. Whereas fungal volatiles promoted root biomass in vitro (Casarrubia et al., 2016; Cordovez et al., 2017; Moisan et al., 2019), our results indicate that root growth rate of *B. rapa* seedlings was not increased in soil upon perception of the fungal volatiles.

**FIGURE 3:**
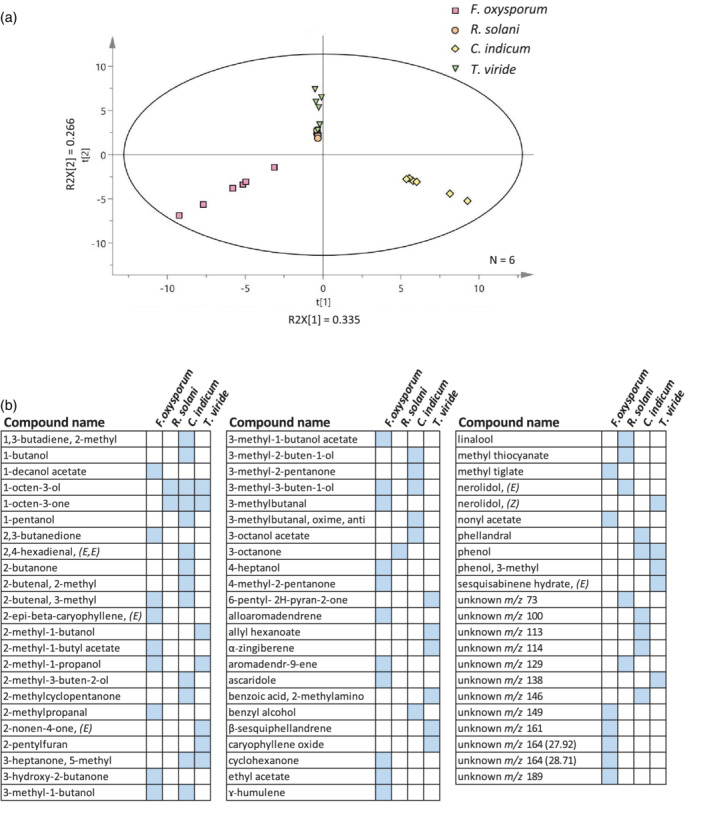
Profiling of volatiles from four fungal species (a) Projection to Latent Structures Discriminant Analysis (PLS‐DA) of volatile organic compounds (VOCs) collected from the headspace of the soil‐borne fungi: *Fusarium oxysporum f.sp. raphani, Rhizoctonia solani, Chaetomium indicum* and *Trichoderma viride*. Grouping pattern of samples according to the first two principal components and the Hotelling's T2 ellipse confining the confidence region (95%) of the score plot. (b) List and presence of the VOCs detected in the fungal VOC headspace of the four fungi [Colour figure can be viewed at wileyonlinelibrary.com]

To begin to unravel which specific volatiles emitted by *R. solani* contribute to the attraction of *B. rapa* roots, we analysed the VOC profiles of all four fungi obtained from our previous study when fungi were cultured on the same media as used in the present study in the absence of plants (Moisan et al., 2019). Comparison of the four VOC profiles showed that *C. indicum* and *F. oxysporum* profiles differ from each other and from the profiles of *R. solani* and *T. viride*, whereas VOC profiles of *R. solani* and *T. viride* were more similar compared to the other two fungi (Figure 3a; PLS‐DA; *R* ^2^ = 0.95; *Q* ^2^ = 0.90, *P*_*CV*_ ANOVA <0.001). Yet, VOC profiles of *T. viride* and *R. solani* were distinct (Figure S2; PLS‐DA; *R* ^2^ = 0.94; *Q* ^2^ = 0.90, *P*_*CV*_ ANOVA = 0.001). Qualitative comparison revealed that only two VOCs, 1‐octen‐3‐ol and 1‐octen‐3‐one, were commonly emitted by *R. solani*, *T. viride* and *C. indicum*, while 6 and 13 VOCs were unique to *R. solani* and *T. viride*, respectively (Figure 3b). The six VOCs unique to *R. solani* include 3‐octanone, linalool, methyl thiocyanate, nerolidol and two unknown VOCs (Figure 3b,c). All these compounds have previously been reported from a microbial source in the mVOC 2.0 database (Lemfack et al., 2017). Surprisingly, none of these VOCs have, to our knowledge, been previously shown to promote root growth, but instead had adverse effects on seed germination and root growth (Table S1). Plant responses may depend on the concentration of the specific VOCs or on a combination of several of these VOCs (Ueda, Kikuta, & Matsuda, 2012). Moreover, the timing and duration of VOCs exposure may determine the phenotypic outcome. Thus, the active compounds or groups of compounds responsible for the attraction of *B. rapa* roots remain to be identified. Studies exploring the production and dynamics of these fungal VOCs in situ, that is, in the presence of roots, will shed more light on these interactions and will help to pinpoint the specific VOCs that mediate these root phenotypic responses. Furthermore, it will be interesting to address these questions in the context of natural ecosystems where myriads of microbial species co‐occur and interact with each other, thus affecting the volatile composition and concentration in soil.

To date, only limited information is available about the mechanisms of root perception to chemicals in soil and the signal‐transduction cascades (Chen, During, & Anten, 2012; Delory, Delaplace, Fauconnier, & Du Jardin, 2016; Sharifi & Ryu, 2018; Tyagi, Mulla, Lee, Chae, & Shukla, 2018). Yet, it is known that roots do not grow randomly through the soil matrix, but can grow toward nutrient patches (de Kroon & Mommer, 2006; Ferrieri et al., 2017; Hutchings & de Kroon, 1994). Different physical and chemical stimuli may influence directional root growth such as a gradient in soil moisture (hydrotropism), light (phototropism) or gravity (gravitropism) (Band et al., 2012; Eapen, Barroso, Ponce, Campos, & Cassab, 2005; Mochizuki et al., 2005). Thus, directional root growth may likely be influenced by volatiles in soil as well (Wenke, Kai, & Piechulla, 2010; Yokawa, Derrien‐Maze, Mancuso, & Baluška, 2014). However, the mechanisms underlying the change in root directional growth are still unknown. The emergence of new technologies that allow non‐invasive root imaging, such as X‐ray or MRI (Atkinson, Pound, Bennett, & Wells, 2019; Nagel et al., 2012; Rogers et al., 2016), can provide new insight in how root direction is affected by microbial volatiles in situ.

Intriguingly, *B. rapa* primary roots were only attracted to volatiles from *R. solani*, which was the only fungus that infected roots of the *B. rapa* accession used (Figure S1). This result was counter‐intuitive as we hypothesised that roots would seek for interactions with beneficial fungi, while avoiding pathogens. The attraction of roots toward volatiles from a pathogenic fungus would provide clear advantages to the fungus, hence our findings suggest a manipulation of the plant growth by the fungus. A larger set of pathogenic and non‐pathogenic fungi need to be tested to support this assumption. Yet, the actual costs and benefits of the volatile emitter and receiver in soil ecosystems remain to be elucidated. Here, we showed that leaf dry weight was not affected upon choice of the primary roots toward the fungal volatiles (Figure S3; Student *t*‐tests; *p* > .05). In conclusion, our novel experimental setup enables investigating the directional growth of roots in soil, thus providing an important step toward deciphering root responses to volatiles emitted by soil‐borne fungi. It raises fundamental questions about the evolutionary ecology of such interactions between roots and fungi, and in particular the concept of “fatal attraction.”

## CONFLICT OF INTEREST

The authors have no conflict of interest to declare.

## Supporting information


**Figure S1.** Disease symptoms on *Brassica rapa* seedlings inoculated with *Rhizoctonia solani* and *Fusarium oxysporum*
**Figure S2.** PLS‐DA of VOCs collected from the headspace of *Trichoderma viride* and *Rhizoctonia solani*
**Figure S3.** Leaf dry weight of *Brassica rapa* upon choice of the primary root toward the control or the fungal volatiles
**Table S1.** List of VOCs emitted solely by *Rhizoctonia solani*, and their reported effects on plants and soil organisms
**Data S1.** Protocol used to test the pathogenicity of the four fungi on *Brassica rapa*
