# Supplementary material for: Volatiles from soil‐borne fungi affect directional growth of roots
Source: Plant Cell Environ. 2020 Sep 30;44(1):339–45. doi: 10.1111/pce.13890 (PMC7821104; doi:10.1111/pce.13890)
Supplement: Supplementary file 1 — Figure S1. Disease symptoms on Brassica rapa seedlings inoculated with Rhizoctonia solani and Fusarium oxysporum Figure S2. PLS‐DA of VOCs collected from the headspace of Trichoderma viride and Rhizoctonia solani Figure S3. Leaf dry weight of Brassica rapa upon choice of the primary root toward the control or the fungal volatiles Table S1. List of VOCs emitted solely by Rhizoctonia solani, and their reported effects on plants and soil organisms Data S1. Protocol used to test the pathogenicity of the four fungi on Brassica rapa [file PCE-44-339-s001.docx]

## ***Plant, Cell & Environment* Supporting Information**

**Volatiles from soil-borne fungi affect directional growth of roots**

Kay Moisan, Jos M. Raaijmakers, Marcel Dicke, Dani Lucas-Barbosa, Viviane Cordovez

The following Supporting Information is available for this article:

**Supplementary Data** **S1**: Protocol used to test the pathogenicity of the four fungi on *Brassica rapa*

**Supplementary Figure** **S1**: Disease symptoms on *Brassica rapa* seedlings inoculated with *Rhizoctonia solani* and *Fusarium oxysporum*

**Supplementary Figure** **S2**: PLS-DA of VOCs collected from the headspace of *Trichoderma viride* and *Rhizoctonia solani*

**Supplementary Figure** **S3**: Leaf dry weight of *Brassica rapa* upon choice of the primary root towards the control or the fungal volatiles

**Supplementary Table** **S1**: List of VOCs emitted solely by *Rhizoctonia solani*, and their reported effects on plants and soil organisms

**Data S1.** Protocol used to test the pathogenicity of *Rhizoctonia solani, Fusarium oxysporum, Chaetomium indicum* and *Tricherderma viride* on *Brassica rapa.*

To determine the pathogenicity of the four fungal strains on *B. rapa*, 1-week-old *B. rapa* seedlings were inoculated with the fungi, and disease incidence was recorded after two weeks. For this, surface-sterilised seeds were sown in plastic pots (6 x 6 x 7 cm) filled with autoclaved sand supplemented with Hyponex nutrient solution, and kept in a growth cabinet (25 ± 1 °C; 16:8 L:D; 70 ± 5% R.H.). For *R. solani*, an agar plug with mycelium was buried in the sand directly in contact with the roots. For the other three fungi, *B. rapa* plants were up-rooted, and roots were incubated for 3 min in a solution of conidia suspended in sterile demi-water (10^6^ conidia ml^-1^). Seedlings were subsequently replanted in sand. Control plants were inoculated either with a plug of sterile agar or by dipping roots into sterile demi-water. After the fungal inoculation, pots were kept inside trays with transparent lids for one week to ensure high humidity, favourable to the fungal infection. Each inoculation was replicated six to seven times. Two weeks after the inoculation, plants were harvested and disease symptoms (*e.g.* lesions, discoloration vascular tissue, wilting) were recorded in the aerial tissues and in the roots, the latter by using a binocular (Leica Microsystems B.V., Son, The Netherlands). Additionally, dry weights of roots and leaves were measured by drying plant tissues at 70 °C for 3 days. Root and leaf dry weights of fungus-inoculated and control (non-inoculated) plants were compared using One-Way ANOVA and Two-Sample Student’s *t*-tests (α = 0.05).

**Figure S1. (a)** Leaf and **(b)** root dry weight (mean ± SE) of two week-old *Brassica rapa* plants inoculated either with a conidia suspension of *Fusarium oxysporum, Chaetomium indicum* or *Trichoderma viride,* or inoculated with mycelium of *Rhizoctonia solani,* and their respective controls; **(c)** Photographs of the aboveground tissues and root tips of control plants and plants inoculated with *R. solani*; **(d)** photographs of root cross sections of control plants and plants inoculated with *F. oxysporum*. Controls were either inoculated with sterile demi-water (used to suspend the fungal conidia) or with a plug of sterile agar (used to culture *R. solani* mycelium). Differences of dry weight were tested with one-way ANOVA and Student *t-*tests, and asterisks indicate statistical differences (*P* < 0.05; NS: *P* > 0.05).

**Figure S2.** Projection to Latent Structures Discriminant Analysis (PLS-DA) of volatile organic compounds (VOCs) collected from the headspace of *Rhizoctonia solani* and *Trichoderma viride*. **(a)** Grouping pattern of samples according to the first two principal components and the Hotelling’s T2 ellipse confining the confidence region (95%) of the score plot; **(b)** Loading plot showing the contribution of individual VOCs to the first two principal components.

**Figure S3.** Leaf dry weight (mean ± SE) of seven day-old *Brassica rapa* seedings upon choice of the primary root towards the control or fungal volatiles. The fungi tested were *Fusarium oxysporum f.sp. raphani, Rhizoctonia solani, Chaetomium indicum* and *Trichoderma viride.* Differences of leaf dry weights and root:leaf ratios were analysed with Student *t*-tests (NS: *P* > 0.05).

| **VOC** | **Receiver** | **Effects** | **References** |
| --- | --- | --- | --- |
| 3-octanone | *Arabidopsis thaliana* | Leaf bleaching at high concentrations | Splivallo, Novero, Bertea, Bossi, & Bonfante (2007) |
|  |  | Reduction of root length |  |
|  |  | No effect on plant growth at low concentrations | Cordovez et al*.* (2017) |
|  |  | Phytotoxic effects at high concentrations |  |
|  |  | Inhibition of root germination and growth | Lee, Hung, Schink, Mauro, & Bennett (2014) |
|  | *Gossypium hirsutum* (cotton) | Inhibition of root elongation | Bradow (1993) |
|  |  |  |  |
| methyl-thiocyanate | *Lactuca sativa* (lettuce) | Possible inhibition of germination | Brown, & Morra (1996) |
|  | *Rhizoctonia solani* | Inhibition of growth | Ossowicki, Jafra, & Garbeva (2017) |
|  |  |  |  |
| linalool | *Steinernema* and *Heterorhabditis* nematodes | Positive or negative effects on chemotaxis | Laznik, & Trdan (2013) |
|  | *Hordeum vulgare* (barley) | Increase of root elongation | Wardle, & Short (1982) |
|  | *Lactuca sativa* (lettuce) | Inhibition of seed germination at high concentrations |  |
|  |  |  |  |
| nerolidol | *Hordeum vulgare* (barley) | Reduction of root elongation at high concentrations | Wardle, & Short (1982) |
|  | *Lactuca sativa* (lettuce) and *Lepidium sativum* (cress) | Inhibition of seed germination |  |
|  |  |  |  |

**Table S1**. List of unique volatile organic compounds (VOCs) identified in the headspace of *Rhizoctonia solani,* and their reported effects on plants

**References**

Bradow, J.M. (1993). Inhibitions of cotton seedling growth by volatile ketones emitted by cover crop residues. *Journal of Chemical Ecology,* *19*(6), 1085-1108.

Brown, P., & Morra, M. (1996). Hydrolysis products of glucosinolates in *Brassica napus* tissues as inhibitors of seed germination. *Plant and Soil,* *181*(2), 307-316.

Cordovez, V., Mommer, L., Moisan, K., Lucas-Barbosa, D., Pierik, R., Mumm, R., … Raaijmakers, J.M. (2017). Plant phenotypic and transcriptional changes induced by volatiles from the fungal root pathogen *Rhizoctonia solani*. *Frontiers in Plant Science,* *8,* 1262.

Laznik, Ž., & Trdan, S. (2013). An investigation on the chemotactic responses of different entomopathogenic nematode strains to mechanically damaged maize root volatile compounds. *Experimental Parasitology,* *134*(3), 349-355.

Lee, S., Hung, R., Schink, A., Mauro, J., & Bennett, J.W. (2014). *Arabidopsis thaliana* for testing the phytotoxicity of volatile organic compounds. *Plant Growth Regulation,* *74*(2), 177-186.

Ossowicki, A., Jafra, S., & Garbeva, P. (2017). The antimicrobial volatile power of the rhizospheric isolate *Pseudomonas donghuensis* P482. *PLoS One,* *12*(3), e0174362.

Splivallo, R., Novero, M., Bertea, C.M., Bossi, S., & Bonfante, P. (2007). Truffle volatiles inhibit growth and induce an oxidative burst in *Arabidopsis thaliana*. *New Phytologist,* *175*(3), 417-424.

Wardle, K., & Short, K.C. (1982). Effects of isoprenoid alcohols and fatty acids on root elongation, germination, and their association with stomatal activity. *Biochemie und Physiologie der Pflanzen,* *177*(3), 210-215.
